# Is Lithium a Micronutrient? From Biological Activity and Epidemiological Observation to Food Fortification

**DOI:** 10.1007/s12011-018-1455-2

**Published:** 2018-07-31

**Authors:** Daria Szklarska, Piotr Rzymski

**Affiliations:** Department of Environmental Medicine, Poznan University of Medical Sciences, Rokietnicka 8, 60-806 Poznań, Poland

**Keywords:** Lithium, Suicide, Micronutrient, Fortified food

## Abstract

Lithium compounds have been widely used in psychopharmacology, particularly in the treatment of bipolar disorder. Their normothymic and neuroprotective properties when used at high doses have been well established. However, a number of observations suggest that environmentally relevant lithium doses may also exert beneficial health effects, leading to a decrease in the rate of suicides and levels of violence. Despite the fact that this element is not officially considered to be a micronutrient, some authors have suggested provisional recommended intakes set at 1000 μg/day for a 70-kg adult (14.3 μg/kg body weight). The present paper reviews the biological action of lithium, its bioavailability and metabolism, and content in different foodstuffs and water. It also assesses epidemiological data on potential correlations between lithium intake and suicide rate as well as examines the concept of fortifying food with this element as a strategy in the primary prevention of mood disorders and pre-suicidal syndrome.

## Introduction

Lithium (Li), discovered in 1817, is a naturally occurring metal in the earth’s crust (0.0017%) which in the solid phase is the least dense of all elements. In the form of carbonate (Li_2_CO_3_), it has become one of the most frequently used pharmaceuticals in psychiatric treatment owing to its normothymic effects. Since 1949, it has been increasingly used in the treatment of bipolar disorder (BD) [1]. In circulation for over half a century, it has outlived a variety of psychotherapeutic trends and it is still recommended especially in the treatment of acute mania and manic episodes [2, 3]. As has also been shown, therapy with Li can reduce the risk of suicide, both in patients with BD and with schizophrenia [4–6].

Therapeutic, oral doses of Li are usually within the limits of 600–1200 mg Li_2_CO_3_ per day (containing 113–226 mg of elemental lithium) [7]. Interestingly, however, Li may reveal a stabilizing effect at much lower doses, even at concentrations found naturally in the environment. A number of observations indicate a lower percentage of suicides in populations that consume water with a higher content of ionic Li [8–13]. It should be noticed that suicide currently contributes to nearly 800 thousand deaths annually around the world [14]. The highest suicidal rates were found in Europe although the epidemiology is known to vary across countries [15]. In most cases, suicides are associated with the occurrence of diseases and mental disorders, including BD [16].

If the beneficial normothymic effect of lithium could be achieved at lower doses (which are much safer) than those used therapeutically, increasing its dietary intake would offer a simple and successful approach to the adjunctive prevention of the incidence of mental disorders and a reduction in suicide attempts. It is not surprising, therefore, that in recent years the modern psychiatry has heard voices calling to consider an introduction of the food fortified with lithium similarly to the model of table salt iodization [17, 18]. So far, however, no food products enriched in lithium have been introduced into the sales market although some research in this regard has been already conducted.

The aim of this paper was to review the normothymic role of dietary Li and possibility to increase its intake by food supplementation on the basis of its biological properties, currently established consumption in various populations, bioavailability and metabolism, and observations from epidemiological studies linking Li with decreased suicidal rate.

## Biological Effect of Lithium

The biological effects of Li in humans are known mostly from the studies in patients with BD; lithium salts are the gold standard in the treatment of this disease [19, 20]. The normothymic action of Li appears to result from its impact on intracellular neurotransmission, and a key location of this action is the central nervous system [3] (Table 1).

**Table 1:** Interaction of Li with selected targets and its effects on cellular level

Target	Mechanism of Li action	Result
G proteins	Inhibition	Adenyl cyclase inhibition Inhibition of PKA Decrease in cAMP concentration
PI3K	Activation	Akt-1 activation GSK3β inhibition
IMP IPP	Inhibition	IP3 inhibition (leading to Ca2^+^ regulation) DAG inhibition (leading to PKC inhibition) GSK3β inhibition
Akt/PKB	Activation	GSK3β inhibition Inhibition of pro-apoptotic factors Decrease in expression of p53 and Bax proteins
GSK3β	Inhibition	Glycogen synthase activation Nuclear factor-κB activation Inhibition of pro-apoptotic factors
CREB	Inhibition	Increased expression of BDNF Increased expression of dynorphins

*Akt/PKB*, RAC-alpha serine/threonine-protein kinase; *BDNF*, brain-derived neurotrophic factor; *CREB*, cAMP response element-binding protein; *DAG*, diacylglycerol; *GSK3β*, glycogen synthase kinase 3 beta; *IMP*, inosine monophosphate; *IP3*, inositol trisphosphate; *IPP*, inositol polyphosphate 1-phosphatase; *PI3K*, phosphatidylinositol-4,5-bisphosphate 3-kinase; *PKA*, protein kinase A; *PKC*, protein kinase C

Li penetrates the interior of the cell mainly by simple diffusion through voltage-dependent sodium channels according to the concentration gradient. Its ions can easily pass through these channels as their permeability is almost equal to that of sodium; the ionic radius of anhydrous Li is less than the radius of sodium and very similar to that of anhydrous magnesium [21]. As observed, the intracellular concentration of Li is usually significantly lower than that in extracellular fluid, due to its displacement from the cell by sodium-lithium counter-transport (SLC). Regulation of the Li clearance rate from the cell has a crucial impact on its therapeutic effects in the course of treatment of various mental alterations, and the SLC mechanism is most likely weakened in the case of affective disorders [3, 22].

The biochemical mechanism of Li action seems to be multifactorial and interdependent with the function of various enzymes, hormones, and vitamins [23]. Numerous studies conducted so far on the exact mechanism of its function in the human body still leave many gaps yet to be fully elucidated [1, 3, 7]. It is possible that the action of Li^+^ ions in cells is based on competition with Na^+^ and Mg^2+^ ions, resulting from the similarity of their atomic radius. It is also postulated that the key to the therapeutic effect of Li lies in the inhibition of enzymes, dependent on the above cations, which regulate intracellular processes and participate in specific nerve transmission pathways. The synthesis and release of neurotransmitters in the cell membrane and the entire cellular metabolism can therefore be modified by the action of Li [3, 7, 24, 25].

It has been shown that Li restores the level of Na^+^ ions and regulates the activity of sodium-potassium ATP-ase, stabilizes the system of secondary relays, and regulates intracellular signal cascades that are dependent on cAMP and Ca^2+^ [26]. Intracellular accumulation of Li results in the replacement of Na, which in turn reduces intracellular Ca^2+^ concentration, inhibits release, and facilitates the uptake of major transmitters: noradrenaline, serotonin, and dopamine [20]. As observed, Li modulates the activity of glutamate, dopamine, serotonin, gamma-aminobutyric acid, acetylcholine, and glycine [3, 20]. It is also known that it can regulate intracellular processes stimulated by synaptic neurotransmitters by acting on phosphatidylinositol and adenylyl cyclase systems [25, 27–29]. It has also been shown to inhibit the activity of glycogen synthase kinases, associated with cell proliferation, metabolism, and apoptosis [7, 20, 30]. In addition, it enhances the expression of protection factors, such as the brain-derived neurotrophic factor, its receptor, and the BAG-1 factor interacting with the bcl-2 protein [25, 28].

By modulating the expression of clock genes (*TIMELESS*, *ARNTL1*, *PER3*, *NR1D1*, *CLOCK*), Li can resynchronize circadian rhythms. It can also normalize the functioning of the hypothalamo-pituitary-adrenal axis by affecting the expression of corticotropins in the adrenal glands [20, 31–33].

According to Schrauzer (2002), the stimulation of vitamin transport, particularly B12 and folic acid to the brain, may constitute one of the normothymic mechanisms of Li action because these vitamins are known to affect parameters related to mood [23]. This is however controversial as some studies reported negative results in this respect and indicated a decrease in serum B12 levels under the influence of Li therapy [34].

The therapy with Li has also been shown to increase density of the gray matter and increase the size of the amygdala and hippocampus. Li is also known to stimulate the production of neural stem cells and has protective effects against oxidative stress and its consequences [25, 35].

An equally interesting aspect of the action of Li is its effect on morphological changes in the blood, resulting in modulation of the immune system response. It is suggested that this element has a complex immunomodulating effect, including suppressor activity and interactions between different classes of white blood cells [7]. As shown in clinical trials, Li induces granulocytosis [36] and lymphopenia [37] but increases the immunological activity of monocytes, NK cells, and lymphocytes, particularly increases the synthesis of IgG and IgM immunoglobulins by B cells [7, 38–40].

The wide spectrum of Li action partially supports its potential role as a micronutrient although it should be noted that the vast majority of the above observations were made when using high, therapeutic doses of this element. It is not yet known whether similar effects and to which extent may occur in the case of the trace doses in which lithium occurs in food and drinking water. Currently, there is no key biological function of Li, without which a lifecycle of living organisms could not be completed [41].

## Lithium Intake, Bioavailability, and Metabolism

The estimations of daily intake of oral Li are very diverse. It can vary, depending on its availability in the environment and in food products, from several to several thousand micrograms per day [23, 42, 43]. As proposed by Schrauzer (2002), the daily requirement for Li is 1000 μg/day for a 70-kg adult (14.3 μg/kg body weight). According to other authors, this is a cautious estimate, not reflecting individual differences that may require even higher consumption to maintain optimal health [17]. However, one should bear in mind that Li is not considered officially as a micronutrient; thus, these recommendations are only provisional and cannot formally be used in dietary practice [23].

Due to the uneven distribution of Li in the Earth’s crust, its estimated consumption in populations of various regions of the world is highly diverse [23]. In Europe, the Li intake is likely low. As shown, its average consumption in whole-day food intake of Polish students amounted to only 10.7 μg [44], while among adults of Belgium, it was estimated at a mean level of 8.6 μg [42]. Interestingly, it was found that a significantly higher Li supply is observed in the autumn-winter period (November–March) than that during the spring-summer season (April–July), which indicates the role of seasonal products in total intake of this element—the association is yet to be fully explored [44].

When supplied in the form of soluble salts, Li is absorbed virtually entirely in the small intestine through sodium channels and evenly distributed in the body, although differences in its concentrations in tissues, plasma, and the brain have been detected [10, 23, 35, 45]. It is likely that the absorption process can be modified depending on the other components of the diet; however, a thorough study of chemical compounds that increase and reduce the absorption of Li in the digestive system requires more detailed investigation [44].

Elimination of Li from the body occurs within 24 h after its oral intake and is facilitated by the kidneys. To a small extent (2–3%), it is also excreted with feces and sweat [23, 45, 46]. The rate of elimination depends on its concentration in plasma, which is proportional to its daily intake. The amount of Li excreted with urine serves as an indicator of the supply of this element [10, 44]. In addition, Li excretion depends on the glomerular filtration rate, and can therefore be reduced with age and in renal disease (e.g., chronic renal failure) [10]. Typical range of Li concentration falls within 4.6–219 μg/L limits [44, 47], and according to some observations, it remains in significant relation to its concentration in the consumed water [10].

Dietary factors, stress, and exposure to exotoxic factors that increase the level of cortisol and other stress hormones affect the physiological demand for a whole range of water-soluble micronutrients (e.g., magnesium, zinc, B vitamins), and probably also for Li [17]. A low-sodium diet, dehydration with loss of electrolytes, edema, as well as the use of antihypertensive drugs (angiotensin-converting enzyme inhibitors, beta blockers, verapamil, and others) and non-steroidal anti-inflammatory drugs (excluding aspirin), reduce the clearance of Li [46, 48]. Under normal conditions, close to approximately 80% of Li is reabsorbed by renal tubules. Increased resorption of Li ions in the renal tubules during psychiatric treatment with increased Li doses is associated with the risk of toxic effects [10, 46, 48], while an increased intake of sodium, xanthines (theophylline and caffeine), nifedipine, and carbonic anhydrase inhibitors (e.g., acetazolamide) increases the excretion of lithium. They can therefore increase the demand for this element. The clearance of Li has also been shown to be increased during pregnancy [17, 46].

## Lithium in Food Products

The main sources of Li in the diet are cereals, potatoes, tomatoes, cabbage, and some mineral waters [44]. It may also be found in some spices such as nutmeg, coriander seeds, or cumin; however, their share in the total supply of this element is negligible in many geographic regions [49]. As estimated, cereal grains and vegetables can cover from 66 to over 90% of the daily Li consumed. The rest comes from food of animal origin and from drinking water [13, 23, 35, 45]. Mushrooms growing in forests are rather a poor source of this element, and cultivated forms can be almost devoid of it if it is present at low content in overgrown substrate [50, 51]. It can be expected that a vegetarian diet that is particularly rich in grains and vegetables will provide more Li than a diet that includes intake of animal proteins. However, this may differ significantly depending on geographical location due to the uneven distribution of Li in the Earth’s crust and the fact that the content of this element in plants depends on its content in the ambient environment [23]. The comparison of Li levels in different foodstuffs is summarized in Table 2.

**Table 2:** Mean lithium content (μg/g dry weight) in different foodstuffs

Cereals	4.4
Fish	3.1
Mushrooms	0.19
Vegetables	2.3
Meat	0.012
Dairy products	0.5
Nuts	8.8

The content of Li in various types of teas was also studied, as tea is commonly drunk all over the world [52]. The lowest mean concentration was found in green tea infusion (0.19 μg/g per dried tea), slightly higher in black tea infusion (0.40 μg/g of dry tea leaves), and the highest in the infusion of red tea (0.64 μg/g of dry tea leaves). Excluding Li that is contained in tap water, a 0.25 L infusion of black, green, and red tea can provide respectively 0.58–1.35 μg/g, 0.07–0.53 μg/g, and 0.72–1.70 μg/g Li [52]. The content of Li in coffee and other beverages (e.g., soft drinks) has so far not been investigated.

None of the studies performed so far have directly examined the potential relationship between Li contained in solid food and mental health, although some investigations indicate a beneficial effect of dietary Li supplementation on mood [53]. Some authors have suggested that optimal Li intake may have a protective effect on the nervous system and have a positive effect on mental health, through the anti-inflammatory and antioxidant effects as well through the regulation of the metabolism of nervous system [35].

The oral intake of approximately 0.5–3 mg of lithium daily results in maintaining its serum concentration at the level of 7 to 28 μg/L, although in some individuals the concentrations were found to be close to 0 [23, 35]. A 2-week supplementation of Li at a dose of 1000 μg (5320 μg Li_2_CO_3_) can raise its serum concentrations of lithium from almost 0 up to 20 μg/L. It is worth noting that this is a much lower dose than those given in the treatment of mental disorders [7, 35].

The two most well-known low-dose forms of Li that are readily available over the counter (OTC) are orotate and aspartate. Due to their stability, they are absorbed, transferred in the intestinal lumen, and transported to the cells mostly in a unionized form. In turn, the pharmacological forms of Li—carbonate and citrate—are easily ionized, generating Li ions outside the interior of the cell, as a result of which their absorption by sodium channels is less effective [17].

## Water as a Source of Lithium

Li is a naturally occurring element in surface waters, mainly in its ionic form [54]. Its concentrations, depending primarily on the processes of weathering of mineral rocks [19, 55], differ depending on geographical region [23, 56] and are clearly correlated with its natural resources in a certain region [10]. The available literature does not provide data on the variability of Li concentrations in water depending on time; however, taking into account its chemical properties in aqueous solutions, it can be considered as a relatively constant value. The small radius of Li^+^ ions and their high hydration are the properties that make it possible to assume that they are stable, and do not react chemically with other compounds during aeration, sand filtration, or in either the distribution system or water installations [4]. Therefore, Li, in various concentrations, can be detected in drinking water [57–59]. However, its assessment is not a part of the standard analysis of drinking water. In Europe, there are no legal requirements for monitoring Li levels in surface and drinking water, and no guideline levels were established [13, 45]. It does not appear, however, that the Li contained in water may undergo significant bioaccumulation and its environmental toxicity is low if any [4].

The typical Li concentrations in freshwaters fall in the range of 1 to 10 μg/L in surface waters, whereas in seawater they usually range from 140 to 200 μg/L [23, 60, 61]. Gaillardet et al. (2003) noted that the water levels of Li in rivers account for 0.16 to 4.5 μg/L [62]. Concentrations reaching approximately 200 μg/L were found in drinking water in selected regions of the USA (Texas), Greece, Japan, England, and Italy [63–65]. In comparison with the medicinal doses of 600–2400 mg Li_2_CO_3_/day (113–452 mg Li/day), concentrations occurring in surface and underground waters are very low [55, 66, 67]. Even though the concentrations of Li in some regions of the world reach as much as 5.2 mg/L [68], in tap water in Europe, they generally reach several dozen micrograms per liter [13, 68]. Daily intake of water at the level of 2.0 L would therefore provide only a fraction of the percentage of a typical therapeutic dose of Li-based salt.

Some studies have demonstrated that bottled water from different manufactures can reach high concentrations of Li [67]. In one Slovakian product, a Li concentration reached as much as nearly 10,000 μg/L. The mean concentration of Li in European bottled waters, however, was estimated at 0.94 μg/L [67]. The mean Li content in tap water and bottled water in Scandinavia (Norway, Sweden, Finland, Iceland) accounted for 0.54 and 0.64 μg/L, respectively [69]. Długaszek and Połeć (2012) reported 2.1–14.9 mg/L of Li in spring and medicinal waters produced in Poland with the higher values observed for the latter [70]. In German, mineral waters were reported to contain 1.5–1320 μg/L of Li [71]; while in Romania, the highest concentration was as low as 0.0719 μg/L [72]. These differences in Li concentrations are likely caused by different ambient Li contents in various geographical regions.

It was preliminarily suggested that a high concentration of Li in drinking water and the resulting increased daily intake of this element in the diet of the inhabitants of some world regions may potentially have a toxic effect on the human body [4, 73, 74]. This hypothesis would however require further research. Regions such as northern Chile [75], northern Argentina [56], and selected regions of Austria (around Graz, in other regions, concentrations are at the level of several dozen μg/L) [10] were designated to have high levels of Li (exceeding 1000 μg/L) in drinking water. It has so far been observed that exposure to such levels of Li in drinking water during pregnancy may disturb calcium homeostasis, in particular by affecting the metabolism associated with vitamin D [74].

## Lithium in Drinking Water and the Risk of Suicide

Clinical and epidemiological data point to specific properties of Li in prevention of suicidal behavior, which are at least partially independent of its mood-normalizing effect [65]. Li is a well-known element of normothymic action and has been used as a part of preventive treatment of patients with suicidal tendencies, as confirmed at the level of meta-analyses [56, 68, 76–78] and randomized placebo-controlled clinical trials [79, 80].

Number of studies indicate a negative correlation between concentration of Li naturally occurring in water and mortality rate due to suicide [7–9, 11, 12, 57, 63, 64, 81]. This relationship is somewhat surprising, as the doses of Li used in therapy are several times higher than naturally occurring in the environment [68]; however, this association has been confirmed in different latitudes on various population groups (Table 3).

**Table 3:** Association between lithium concentration in water and suicide rate as observed in epidemiological studies (+ positive correlation; − negative correlation; *x* no correlation)

Location	Lithium concentration (μg/L)	Correlation	Sex differences	Reference
USA (Texas)	0–160 (C)	–	Not available	[8]
Japan (Oita)	0.7–59	–	Only in men	[9]
Austria	33–1300	–	Only in women	[10]
England	0–21	x		[58]
USA (Texas)	2.8–219	–	Not available	[63]
Greece	0.1–121	–	Not available	[12]
Japan (Aomori)	0–12.9	–	Only in women	[11]
Japan (Kyushu)	0–130	–	Only in men	[81]
Italy	0.11–60.8	–	Only in women	[64]
Japan (Hokkaido and Kyushu)	0.1–43	–	Only in men	[82]
Lithuania	0.48–35.5	–	Only in men	[13]
Denmark	0.6–30.7	+		[55]

For the first time, an inverse correlation between the concentration of Li in drinking water and suicide rates was recorded in the USA. Differences in the incidence of suicides, murders, and rapes were reported in the population of 27 counties in Texas which were divided according to Li concentration into three groups: high, 70–160 μg/L; medium, 13–60 μg/L; and low, 0–12 μg/L [7]. Research carried out in 226 counties of Texas and 34 Greek prefectures indicated the inverse correlation of lithium concentration in water and suicide rates; however, in this study, these effects were also not considered separately depending on the sex [12, 63]. Such differences could be potentially expected as some data from therapeutic use of Li indicates that men may be more responsive to Li [83, 84]. It is also postulated that Li may exert its antisuicidal effects by lowering testosterone levels [85].

The first study to potential differences between male and female in response to trace Li in drinking water was conducted in the Oita prefecture of Japan. As reported, the concentration of Li (at a level of 0.7–59 μg/L) was inversely correlated with the frequency of standardized mortality rates (SMR) only among men [9]. These results were later confirmed by a study covering 274 municipalities of Kyushu Island in Japan [81] and confirmed by another, larger survey [82]. In Europe, this type of study was carried out for nine Lithuanian cities. A significant correlation was found between the concentrations of lithium (ranging from 0.5–35 μg/L) in water and SMR in the whole population and in the group of men. The observed concentrations of Li ranged from 0.5 to 35.5 μg/L [13].

Contrary to this, a study carried out in Austria [10] and Japan [11] indicated a relationship between the concentration of lithium in drinking water (0–82.3 and 0–13 μg/L, respectively) and the SMR in the general population and among women, and did not detect any similar association in men. Similarly, a study conducted in the Aomori prefecture of Japan also linked Li higher concentrations with lower SMR in women, with no such correlation observed for men [11]. In Italy, the concentration range in drinking water from 145 areas was in turn from 0.1 to 61 μg/L. The results were analyzed using SMR based on data from 1980 to 1989, 1990 to 1999, and 2000 to 2011. An SMR correlation with the tested lithium concentrations was found in the first time interval for the general population and for women, while in the remaining time intervals, a statistically significant relationship was found only for the suicide rate among women [64].

The study conducted in the eastern part of England, where Li concentrations ranged up to 21 μg/L, did not find them to be significantly associated with SMR, regardless of the sex of the studied population [58]. Surprisingly, in Denmark, despite a similar geographical location and similar ranges of Li concentrations (0.6 to 31 μg/L), the frequency of suicides has been shown to increase with increasing Li concentration in drinking water [55].

The above studies indicate differences in responses to environmental concentrations of lithium depending on gender although contradictory results have been reported with some studies suggesting antisuicidal action in women, others finding it only in men, and some not reporting such associations or reporting potentially pro-suicidal correlation. These differences may be due to geographically diversified response to Li but may also result from limitations of conducted studies. Particularly, the pioneering research in this field lacked the results of weighted variables used in the analyses, as well as reference to potentially disturbing socioeconomic factors. The risk of error in the majority of conducted studies may increase by the use of data on overall lithium concentration in a given region instead of basing such analysis on the individual Li intake levels [9, 11, 12, 58, 63, 64, 68, 82]. Knudsen et al. (2017) were the first to study the link at the individual level, using prospective data collected in Danish registers, and found no antisuicidal effect of Li [55]. It should be remembered that Li concentrations occurring in surface, ground, or underground waters in a particular area, and therefore also in tap water, do not necessarily reflect the intake of this element in the population because the examined individuals may consume bottled mineral water originating from regions distant from the place where they live.

On the other hand, extremely different results may derive from large variations in Li concentrations. In two studies in which no correlation was found, the lowest levels of Li in drinking water were demonstrated at the same time [55, 58]. A small range of concentrations may itself limit a detection of any statistically significant relationship. It can be hypothesized, therefore, that relatively higher concentrations of Li in drinking water (although still much lower from therapeutic doses) may potentially be associated with a reduced frequency of suicides [10, 86]. Further in-depth large-scaled investigations that will consider realistic, individual Li intakes and adjustment for possible confounding factors influencing suicide risk are however required to clarify this hypothesis.

## Potential Strategies to Increase Dietary Lithium Intake

It is hypothesized that a very low Li intake can cause mood worsening and increase impulsiveness and nervousness [84]. This is partially supported by the increased frequency of suicide attempts, homicides, and acts of violence observed among populations within areas with low concentrations of Li in water resources (0–12 μg/L) [8, 57]. One of the hypotheses of the normothymic action of Li in the doses taken with the diet assumes that it may be required for the transport and absorption of vitamin B12 and folates that are involved in neuromodulation and the normal course of biochemical transformations in the central nervous system. Thus, limited intake of Li could also inhibit action of these compounds [17, 23]. The levels of Li in water and in food, and therefore its daily intake in some parts of the world, are low: below the provisional recommendation established by Schrauzer (2002) at 1000 μg [11, 58]. It was therefore suggested that in individuals inhabiting such regions, Li supplementation could be considered [23]. This would be possible either by using food supplements containing lower than therapeutic doses of Li or by introducing additional products enriched in this element, just as salt is commonly enriched with iodine.

According to Goldstein and Mascitelli (2016), an interesting strategy would be to consider the possibility of adding elemental Li to vitamin preparations for adolescents and adults [35]. However, there are supplements containing lithium compounds on the market, e.g., Li orotate, the clinical efficacy of these preparations has not been investigated—only one study has shown a normotomy effect with the use of Li supplementation at a dose of 0.4 mg [17, 53]. Moreover, caution is advised in the use of food supplements; it is important that the consumer be fully acquainted with and follow the information provided by the manufacturer [17, 87]. The literature has already reported a case of mild intoxication manifested by severe nausea and vomiting as a result of an intentional overdose of a Li-containing food supplement by ingestion of 18 tablets with a total dose of 83 mg Li [88].

The risk of intentional abuse of the supplement or accidental poisoning due to interaction in the gastrointestinal tract or as a result of reduced Li of lithium from the body (low-sodium diet, renal failure) also constitutes an obstacle to increasing the intake of this element at a population level [46, 89]. Higher doses of Li could potentially lead to exacerbation of symptoms of some diseases, e.g., psoriasis in sensitive individuals [90]. It is also relevant to consider the possible side effects of low-dose Li on the functioning of the thyroid gland and kidneys, as well as on pregnancy and fetal development, although the risk of low-dose teratogenicity is considered to be relatively low [7, 74].

In reference to the effective supplementation with folic acid (in the prevention of neural tube defects) of breakfast cereals, some authors have proposed the introduction of Li-enriched cereal products [35]. It should be noted that so far no product fortified with Li has been introduced to the market. A method of biofortification of selected mushroom species, including oyster mushrooms (*Pleurotus ostreatus*, *P. eryngii*), the lingzhi mushroom (*Ganoderma lucidum*), and hedgehog mushroom (*Hericium erinaceus*), based on cultivating them on media enriched with the lithium salt, has however been elaborated [43, 94, 95]. In contrast to many plants, cultivation of mushrooms on fortified substrate leads to its uptake by mycelium and translocation to fruiting bodies [43, 91]. The most effective results were observed with the use of Li chloride (LiCl), whose presence in the medium had no significant impact on the growth of the fruit bodies, their morphology and mineral composition, and the accumulated Li was characterized by a higher availability when compared to its carbonate form available in a commercial therapeutic preparation [42, 92, 93]. As estimated, consumption of 100 g dry matter of *H. erinaceus* and *G. lucidum*, grown on a substrate enriched with 1 mM of Li, would cover 69% and 740% of the provisional recommended daily intake (1.0 mg), respectively [43, 93]. However, the possible effectiveness of food fortified with Li in stabilizing mood still requires investigations, first using in vivo model and ultimately, on the level of clinical trials.

The main limitation in fortification of food with Li is associated with little knowledge about the chronic influence of low doses of this element on the human body. It is not officially considered a micronutrient, the minimum level of consumption necessary for maintaining health is unknown and not established [19, 41, 94]. Any changes in this regard would therefore require safety assessment, toxicological tests, and determination on minimum daily intake required to maintain optimal health. Considering a great deal research required to ensure consumer safety, its high costs may negatively impact a success of implementing fortified food on the market.

However, some studies have estimated the daily consumption of Li with food [42, 44], the introduction of fortified products would first require determination of intake of this element in a given population. There are no data on bioavailability of Li from individual foods, and in what chemical form the Li would be the most absorbable. The factors that may modify its absorption from food in the gastrointestinal tract are also poorly elucidated [17, 19]. Finally, it is important that products fortified with Li were not characterized by modified nutritional value and altered appearance (e.g., color), taste, or smell, because such modifications may negatively affect their choice by consumers [95].

## Conclusions

The increasing problem of suicides and mental disorders in developed countries creates an urgent need to develop novel prophylactic strategies to protect mental health. According to numerous observations, the intake of Li, e.g., from drinking water, may negatively affect suicide rates; although in some studies, such correlation has not been demonstrated. Even though Li is not officially considered a micronutrient, according to some authors, it meets the criteria of this group. Considering the potential role of Li in the modulation of the nervous system function, it may be required for normal metabolism and neural communication—this hypothesis, however, requires further in-depth research investigating the mechanisms of action of trace doses of Li. In the light of current knowledge, one cannot explicitly consider the implementation of enrichment of food or drinking water with Li for the general prevention of mental disorders, suicides, or violence. However, Li supplementation in trace doses should be an area of active research given the high suicide rates, epidemiological evidence of protection from trace doses of Li, biological plausibility, and relative safety of Li supplementation at low doses.
